# Recurrence of a congenital diaphragmatic hernia 57 years postoperatively: A case report and review of the literature

**DOI:** 10.1097/MD.0000000000028650

**Published:** 2022-01-21

**Authors:** Kohei Horiguchi, Sang-Woong Lee, Tetsunosuke Shimizu, Jun Arima, Kohei Taniguchi, Seita Hagihara, Koji Komeda, Kazuhisa Uchiyama

**Affiliations:** a Department of General and Gastroenterological Surgery, Osaka Medical and Pharmaceutical University, 2-7 Daigaku-machi, Takatsuki, Osaka, Japan; b Translational Research Program, Osaka Medical and Pharmaceutical University, 2-7 Daigaku-machi, Takatsuki, Osaka, Japan.

**Keywords:** congenital diaphragmatic hernia, laparoscopic surgery, recurrent

## Abstract

**Rationale:**

Postoperative recurrence of congenital diaphragmatic hernia (CDH) in adults is very rare. There is currently no precedent and no established treatment. We encountered a case of CDH which recurred 57 years, postoperatively.

**Patient concerns:**

A 57-year-old man with dyspnea on exertion was referred to our hospital. He had undergone surgery at the same hospital for CDH when he was 46 days old.

**Diagnosis and interventions:**

Laboratory studies, except diagnostic imaging and spirometry, were otherwise within normal limits. He was diagnosed with recurrent CDH based on computed tomography and underwent laparoscopic surgery.

**Outcomes:**

His postoperative course was uneventful, and there was no recurrence on follow-up.

**Lessons:**

We reported our encounter with a case of recurrent CDH, more than 50 years after the initial surgery. When managing diaphragmatic hernias, prompt surgical treatment, with consideration to prior surgical history for CDH, leads to satisfactory results.

## 1 Introduction

Congenital diaphragmatic hernia (CDH) is a condition in which intra-abdominal organs prolapse into the thoracic cavity due to a congenital defect of the diaphragm arising from a developmental abnormality.^[1]^ The first line of treatment is surgery, which involves repair and closure of the hernia portal, and the long-term postoperative course is considered to be good. However, there is still no consensus on which approach yields better results between open or laparoscopic surgical approaches and on whether primary closure or mesh repair is better.

Generally, postoperative recurrence of CDH is believed to be common in early childhood. We encountered a case of recurrent CDH, which manifested 57 years postoperatively that subsequently underwent successful mesh repair. To the best of our knowledge, this is the first report of CDH recurrence noted after such a long duration following the initial surgery. We also included a brief review of relevant literature.

## 2 Case presentation

**Figure 1:**
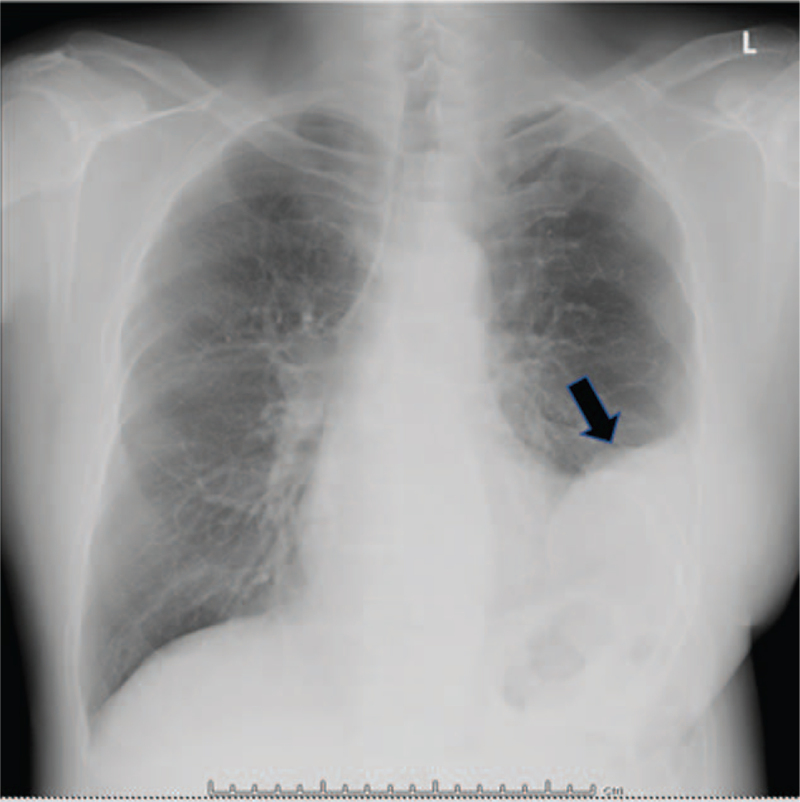
Infiltrative shadow in the left lower lung field upon chest radiography. The black arrowhead indicates that abdominal organs have prolapsed into the thoracic cavity.

**Figure 2:**
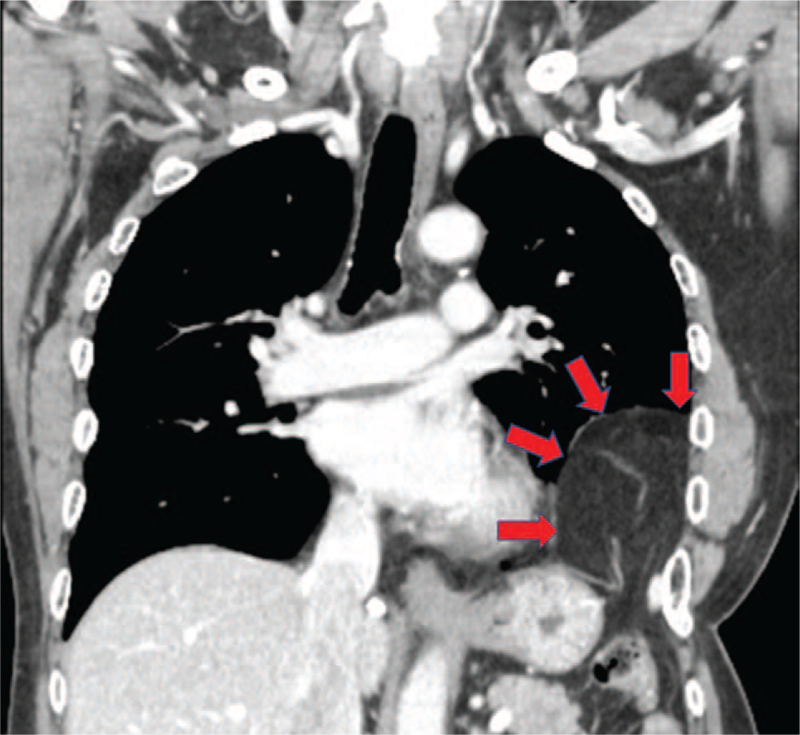
Chest computed tomography angiography (coronal section). The red arrow shows the content of the hernia, suspected to be the greater omentum.

A 57-year-old man with dyspnea on exertion was referred to our hospital. Family, genetic, and psychosocial history were otherwise unremarkable. He had undergone surgery at our hospital for CDH at the age of 46 days. At this time, all laboratory parameters were within normal limits. A chest radiograph showed an infiltrative shadow in the left lower lung field (Fig. 1). Respiratory function test showed mixed impairment; % forced expiratory volume was 61.3% and % vital capacity was 59.5%. Contrast-enhanced computed tomography of the chest showed hernia contents, suspected to be the greater omentum, in the left thoracic cavity (Fig. 2). Accordingly, the patient was diagnosed with recurrent CDH.

**Figure 3:**
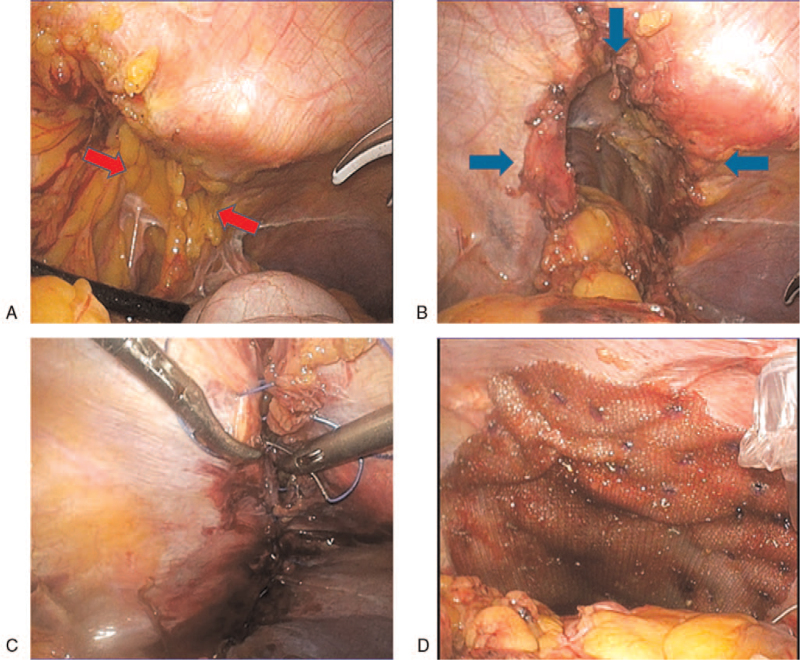
Intra-operative findings. (A) The red arrow shows the greater omentum. The greater omentum herniates into the left thoracic cavity. (B) The area surrounded by the blue arrow is the hernia portal. The size of the hernia orifice is 12 × 3 cm. (C) Direct suture of the hernia orifice using non-absorbable spinous thread. (D) The sutured hernia orifice covered with a mesh.

We decided to perform a laparoscopic hernia repair. Intra-operative findings are shown in Figure 3. The hernial content was the greater omentum (Fig. 3A). After pulling out the hernial content, we detected a 12 × 3 cm hernia sac (Fig. 3B). The diaphragm and the mural peritoneum were sutured with a non-absorbable spinous thread (Fig. 3C). The surrounding tissue was fragile and strained, and primary closure was deemed inadequate; therefore, it was overlaid with Ventlight ST mesh (Fig. 3D).

**Figure 4:**
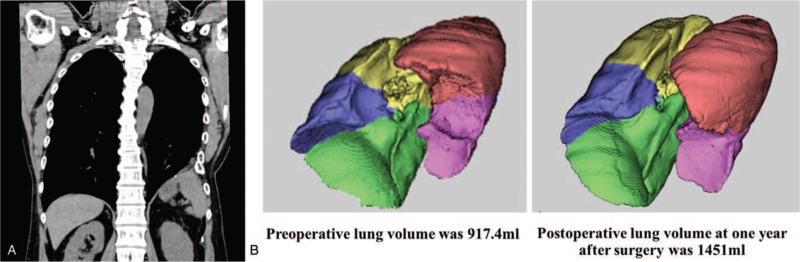
Postoperative chest X-ray and lung volumetry. (A) No recurrence of congenital diaphragmatic hernia on postoperative chest X-ray. (B) The volume of the compressed lung re-expanded from 917 mL to 1451 mL on lung volumetry.

The postoperative course was uneventful, and the patient was discharged 7 days postoperatively after confirming that the left lung had expanded. At the 1-year follow-up visit, physical and radiological examinations showed no signs of recurrence (Fig. 4A), and the volume of the left lung on 3D computed tomography had increased from 917 mL to 1451 mL, signifying recovery (Fig. 4B).

## 3 Discussion

The incidence of CDH is about 1 in 2500 to 4000 live births, and it is diagnosed most frequently in the neonatal period.^[2]^ The pathognomonic finding is prolapse of intra-abdominal organs through the diaphragm into the thoracic cavity on imaging. Prenatal screening may also be used to diagnose CDH.

The first line of treatment is surgical repair. The goal of surgical treatment is to achieve a tension-free repair.^[3]^ If the diaphragmatic defect is small, primary repair may be recommended; if the defective hole is large, patch repair or flap repair should be performed. However, according to some reports, the recurrence rate for the preferred procedure varies from 10% to 20%, and no particular surgical procedure has been established as the most suitable.^[4–6]^ Regarding long-term outcomes of 160 patients with CDH who underwent simple closure of diaphragmatic hernias, Jancelewicz et al^[7]^ observed recurrence in 10% of cases, and most recurrences occurred in infancy, within 2 years after surgery. Of note, our patient experienced recurrence after over 50 years following repair, and we believe this is a very rare case.

In this case, although mild respiratory dysfunction was observed, the patient had not had any respiratory symptoms until consultation. One of the most meaningful lessons we considered was to suspect the recurrence of CDH upon conduct of imaging. To obtain a definitive diagnosis, the following factors should be considered: presence or absence of trauma or abdominal bruise; diaphragmatic laxity; history of abdominal surgery; location of the hernia; and sound knowledge of CDH.

Regarding the above points, since the patient had no history of an automobile accident or abdominal surgery, a traumatic hernia was ruled out. Thereafter, we had to rule out diaphragmatic laxity.^[8]^ We could not obtain details of the patient's surgery at infancy because it was conducted more than 50 years ago. However, we considered diaphragmatic laxity to be unlikely because there was no hernia sac based on current surgical findings. The hernia portal was located posterolateral to the diaphragm, at a location coinciding with the foramen of Bochdalek. This convinced us of the recurrence of a CDH, intra-operatively.

Regarding the recurrence of the diaphragmatic hernia in this patient, we considered 2 probable causes. First, the patient's hobby included the strenuous activity of mountain climbing. Climbing may have increased his intra-abdominal pressure, which may have led to recurrence of CDH. Second, the patient had a long-term smoking history of 37 pack years and had a chronic cough; this may have led to a chronic increase in intra-abdominal pressure and the consequent exertion of pressure on the diaphragm.

A case of diaphragmatic hernia that occurred 18 years postoperatively in a patient who had diaphragmatic laxity in infancy has been reported^[9]^; however, no case of hernia recurrence in a patient after over 50 years post-CDH surgery has been reported. Thus, the appropriate surgical approach was unclear. If there is a diagnosis of adult diaphragmatic hernia, immediate surgery for organ reduction and closure of the hernia orifice are recommended. Open approaches include thoracoscopic, laparoscopic, and combined thoracoabdominal surgery. Recently, there has been an increase in the number of reports of thoracoscopic and laparoscopic procedures. However, no consensus on the choice of approach or surgical technique has been reached. Miyasaka et al^[10]^ recently reviewed the details of 90 cases of adult Bochdalek hernia. According to this report, simple closure and simple closure with mesh were employed for hernia repair in 56.6% and 17.7% of the patients, respectively, despite the availability of endoscopic and open approaches. Further, most patients who underwent hernia repair had satisfactory clinical results. We opted for laparoscopic surgery; however, the priority was to close the hernia via any means.

## 4 Conclusion

Although very rare, CDH recurrence should be considered when managing patients with suspected diaphragmatic hernia. With prompt surgical treatment, our patient recovered adequately and his respiratory function improved.

## Acknowledgments

The authors are grateful for the cooperation of the all staff who were engaged in the patient's treatment. We would like to thank Editage (www.editage.com) for English language editing.

## Author contributions

SW-L, KH, KT, and TS designed the study.

JA, SH, KK, and KU performed the operation and pre-operative care.

KH wrote the paper. TS, JA, KT, and SWL reviewed and revised the manuscript.

KU supervised this report.

All authors approved the final version of this manuscript.

**Writing – original draft:** Kohei Horiguchi, Tetsunosuke Shimizu, Jun Arima, Kohei Taniguchi.

**Writing – review & editing:** Sang-Woong Lee, Tetsunosuke Shimizu, Jun Arima, Kohei Taniguchi, Seita Hagihara, Koji Komeda, Kazuhisa Uchiyama.
